# The Role of the CCR5 Receptor in Neuropathic Pain Modulation: Current Insights and Therapeutic Implications

**DOI:** 10.3390/biomedicines13112650

**Published:** 2025-10-29

**Authors:** Mario García-Domínguez

**Affiliations:** 1 Program of Immunology and Immunotherapy, CIMA-Universidad de Navarra, 31008 Pamplona, Spain; mgdom@unav.es; 2 Department of Immunology and Immunotherapy, Clínica Universidad de Navarra, 31008 Pamplona, Spain; 3 Centro de Investigación Biomédica en Red de Cáncer (CIBERONC), 28029 Madrid, Spain

**Keywords:** CCR5, neuropathic pain, peripheral sensitization, central sensitization, synaptic plasticity

## Abstract

Neuropathic pain, a chronic condition arising from injury or dysfunction of the somatosensory nervous system, is characterized by persistent hypersensitivity and spontaneous pain. The chemokine receptor CCR5 (C-C motif chemokine receptor 5) has recently been identified as a critical mediator in neuroinflammation and neuropathic pain signaling pathways. Expressed on immune cells and neurons, CCR5 regulates immune cell recruitment and activation, thereby contributing to neuronal sensitization and maintenance of pain states. This review examines the currently characterized molecular mechanisms through which CCR5 modulates neuropathic pain pathophysiology and assesses the potential of CCR5 antagonists as novel therapeutic agents for the management of chronic neuropathic pain. Understanding the involvement of CCR5 in pain modulation may facilitate the development of targeted treatments with improved efficacy and safety profiles.

## 1. Introduction

The International Association for the Study of Pain (IASP) defines pain as an “unpleasant sensory and emotional experience associated with, or resembling that associated with, actual or potential tissue damage” [1,2]. This definition highlights that pain is a multifactorial phenomenon that serves as a critical diagnostic indicator, frequently signaling underlying health conditions that require medical intervention [3]. Pain can be classified into distinct categories according to its underlying characteristics, including its origin [4]. Nociceptive pain arises from actual or potential damage to non-neural tissue due to the activation of nociceptors [5]. Nociplastic pain originates from altered nociception in the absence of clear evidence of actual or potential tissue injury that would activate peripheral nociceptors, or without any demonstrable disease or lesion of the somatosensory system capable of causing pain [6]. Finally, neuropathic pain results from a lesion or disease affecting the somatosensory nervous system; however, this pain subtype will be addressed in detail later.

Neuropathic pain is a highly prevalent, chronic, and debilitating condition that arises as a consequence of injury or dysfunction within the somatosensory nervous system [7]. It represents a significant global health burden, affecting an estimated 7–10% of the population, and is associated with reduced quality of life, impaired daily functioning, and substantial socioeconomic costs [8,9,10]. Unlike nociceptive pain, which occurs from actual or potential tissue damage and serves as a protective mechanism by signaling the presence of noxious stimuli, neuropathic pain occurs independently of peripheral injury and frequently manifests as a maladaptive, chronic condition [11,12].

Clinically, neuropathic pain presents with a heterogeneous spectrum of sensory abnormalities. Patients usually report spontaneous pain that occurs without any apparent external trigger, which might manifest as continuous burning, tingling, or aching sensations [13]. In addition, paroxysmal episodes of sharp, shooting, or electric shock-like pain are often observed, reflecting aberrant excitability within nociceptive pathways [14]. Neuropathic pain is also associated with increased responsiveness to noxious stimuli, referred to as hyperalgesia, as well as the induction of pain by normally innocuous stimuli, known as allodynia [15,16]. These sensory disturbances can be further classified into mechanical, thermal, or dynamic subtypes, underscoring the complex and multifaceted nature of the disorder and its impact on somatosensory processing [17]. The complex symptomatology and interindividual variability in clinical presentation highlight the multifactorial nature of neuropathic pain and the need for a deeper understanding of its molecular underpinnings [18].

From a molecular perspective, neuropathic pain is increasingly understood as more than aberrant neuronal firing; it represents a complex neuroimmune disorder in which bidirectional communication between immune cells, glial cells, and neurons orchestrates changes in neuronal excitability and synaptic architecture [19]. Peripheral nerve injury or CNS insult rapidly triggers a cascade of inflammatory and immune responses. Damaged neurons, Schwann cells, and surrounding non-neuronal cells release many pro-inflammatory cytokines (e.g., TNF-α, IL-1β, and IL-6), chemokines (e.g., CCL2, CCL3, and CCL5), and damage-associated molecular patterns (DAMPs) [20,21,22]. These molecules operate as danger signals, activating resident glial populations (primarily microglia and astrocytes) via pattern recognition (PRRs) and cytokine/chemokine receptors [23,24,25]. Activated glial cells, in turn, release additional pro-inflammatory mediators and neurotrophic factors that modulate neuronal activity and synaptic function (such as IL-1β, TNF-α, BDNF, and NGF) [26,27]. In parallel, chemokine gradients and adhesion molecules (e.g., ICAM-1) foster the recruitment and infiltration of peripheral immune cells, including macrophages, T lymphocytes, and neutrophils, into the injured nerve and central pain-processing regions including the spinal dorsal horn and supraspinal centers [21,28]. This long-lasting neuroinflammatory environment promotes profound alterations in neuronal physiology. VGSCs (Nav1.3 and Nav1.7), VGCCs (e.g., Cav2.2), and K^+^ channels show altered expression and biophysical properties, contributing to ectopic firing and hyperexcitability of nociceptive neurons [29,30,31]. Simultaneously, dysregulation of excitatory neurotransmitters such as glutamate and aspartate, along with impaired inhibitory GABAergic and glycinergic signaling, facilitates central sensitization [32,33]. Maladaptive synaptic plasticity, including long-term potentiation (LTP) at dorsal horn synapses, amplifies nociceptive signal transmission and underlies persistent hypersensitivity to both noxious and innocuous stimuli [34].

Among the diverse molecular mediators that orchestrate neuroimmune communication in neuropathic pain, chemokine signaling has emerged as a key determinant of pain hypersensitivity [35]. Chemokines, a highly conserved family of small secreted cytokines (8–12 kDa), are classified into four major subfamilies (CC, CXC, CX3C, and XC) based on the arrangement of conserved cysteine residues, with receptors such as CCR1-CCR10, CXCR1-CXCR7, CX3CR1, and XCR1 mediating their biological effects [36]. These proteins exert numerous roles in the immune and nervous systems, as they modulate the recruitment, migration, and activation of immune cells (e.g., microglia, macrophages, and T cells) at sites of neural injury or inflammation [37]. Beyond their classical immunomodulatory functions, chemokines influence neuronal activity by modulating membrane excitability, synaptic transmission, and the plasticity of pain pathways [38].

The CCR5 receptor has garnered considerable attention for its involvement in regulating immune responses and modulating neuronal excitability [39,40]. CCR5 is a GPCR expressed across diverse subsets of immune cells (such as macrophages, monocytes, and T lymphocytes) [41], along with glial cells (such as microglia and astrocytes) [42,43], and some neuronal populations [44]. Upon binding its endogenous ligands, such as CCL3, CCL4, and CCL5, CCR5 activates several signaling cascades, in particular, the PI3K/Akt, MAPK/ERK, and NF-κB pathways [45,46]. Activation of these pathways controls the recruitment of additional immune cells, boosts glial reactivity, promotes transcription and release of secondary pro-nociceptive mediators (e.g., IL-1β), and supports synaptic modifications in dorsal horn neurons [47]. Collectively, these processes establish a self-perpetuating feed-forward loop of neuroinflammation, glial activation, and neuronal sensitization that drives the initiation and chronicity of neuropathic pain [48,49].

Despite decades of research, current therapeutic options for neuropathic pain remain unsatisfactory. Conventional first-line therapies, such as anticonvulsants (e.g., gabapentinoids) and antidepressants (e.g., amitriptyline), produce limited analgesic efficacy in a proportion of patients [50,51]. Opioids, although usually prescribed, are characterized by limited effectiveness, the propensity for tolerance induction, and an increased risk of dependence [52]. Moreover, several available drugs carry substantial adverse effects, involving sedation, dizziness, gastrointestinal disturbances, and cognitive impairment, which further restrict their long-term utility [53]. These limitations underscore the need for novel therapeutic strategies with improved efficacy that are mechanism-oriented, targeting the cellular and molecular pathways responsible for the maintenance of neuropathic pain.

In the context of neuropathic pain, CCR5 is recognized to exert dual effects on pain regulation (mediating immune responses and influencing neuronal excitability). This role of CCR5 establishes it as a promising therapeutic target, bridging immune and neuronal pathways that underlie chronic pain [54]. Pharmacological blockade or genetic deletion of CCR5 has been shown in experimental models to attenuate glial activation, reduce cytokine and chemokine production, normalize neuronal excitability, and ultimately alleviate pain-like behaviors [55,56]. Indeed, emerging evidence increasingly implicates CCR5 as a pivotal regulator of neuroinflammatory pathways involved in the initiation and maintenance of neuropathic pain. Preclinical rodent models of neuropathic pain employ diverse strategies to mimic distinct clinical phenotypes. Chronic constriction injury (CCI) involves loose ligation of the sciatic nerve, inducing partial nerve damage and persistent mechanical and thermal hypersensitivity [57]. Partial sciatic nerve ligation (PSNL) consists of tight ligation of a segment of the sciatic nerve, producing robust and long-lasting allodynia and hyperalgesia [57]. Chemotherapy-induced peripheral neuropathy (CIPN) is generated by administration of neurotoxic chemotherapeutics, such as paclitaxel or oxaliplatin, causing sensory neuropathy with mechanical and thermal hypersensitivity [58]. Diabetic neuropathy (DPN) is modeled via streptozotocin injection or genetic diabetes, leading to chronic hyperglycemia and progressive sensory deficits, recapitulating key features of human diabetic neuropathy [59]. In view of the convergence of immune and neuronal signaling at the level of CCR5, targeted inhibition of this receptor holds considerable promise for the development of novel mechanism-based therapies for neuropathic pain.

This review provides a comprehensive overview of the molecular and cellular mechanisms through which CCR5 contributes to the initiation and maintenance of neuropathic pain. In addition, the therapeutic potential of CCR5 antagonists (many of which have been developed and evaluated in other clinical contexts, such as HIV-1 infection) is assessed as a strategy for the treatment of chronic pain.

## 2. CCR5: Structure and Physiological Functions

### 2.1. Structural Features and Molecular Signaling Mechanisms of CCR5

The CCR5 is a prototypical member of the class A GPCR family, a large and evolutionarily conserved group of membrane proteins that mediate cell communication in response to extracellular stimuli [60]. Similarly to other G protein-coupled receptors, CCR5 is characterized by seven transmembrane (7TM) α-helical domains organized in a canonical serpentine topology that crosses the lipid bilayer [61]. These helices are interconnected by three extracellular loops (ECL1–ECL3) and three intracellular loops (ICL1–ICL3), creating a structural framework that allows dynamic conformational rearrangements upon ligand binding [62].

At the extracellular level, CCR5 possesses an N-terminal domain rich in tyrosine residues that are frequently sulfated [63], a post-translational modification essential for high-affinity binding to several chemokine ligands, including CCL3, CCL4, and CCL5. In addition, this N-terminus undergoes O- and N-linked glycosylation, which modulates receptor folding, trafficking to the plasma membrane, and ligand specificity [64,65]. The extracellular loops, particularly ECL2, also contribute critically to chemokine recognition and fine-tuning of ligand selectivity, forming a multi-point binding interface that coordinates the chemokine’s core domain with the receptor pocket [66].

**Figure 1:**
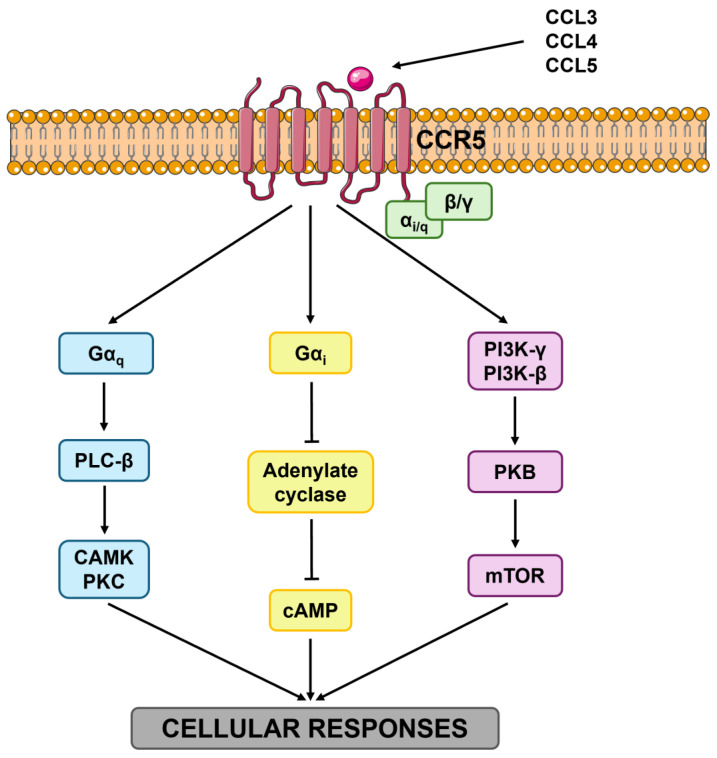
Intracellular signaling pathways mediated by CCR5. Binding of CCR5 ligands (principally CCL3, CCL4, and CCL5) to the CCR5 receptor activates several heterotrimeric G proteins, whose distinct subunits subsequently initiate specific signaling cascades: (1) Gαq stimulates PLC-β, leading to the activation of CaMK and PKC; (2) Gαi inhibits adenylate cyclase, thereby reducing intracellular cAMP levels; (3) the Gβγ subunit activates PI3K-γ/β, which subsequently triggers PKB and the mTOR pathway. Abbreviations: CCR5 (C-C chemokine receptor type 5), CCL3 (chemokine -C-C motif- ligand 3), CCL4 (chemokine -C-C motif- ligand 4), CCL5 (chemokine -C-C motif- ligand 5), Gαq (G protein alpha q subunit), Gαi (G protein alpha i subunit), PLC-β (phospholipase C beta), CaMK (Ca^2+^/calmodulin-dependent protein kinase), PKC (protein kinase C), cAMP (cyclic adenosine monophosphate), PI3K-γ (phosphatidylinositol 3-kinase gamma), PI3K-β (phosphatidylinositol 3-kinase beta), PKB (protein kinase B), and mTOR (mammalian target of rapamycin).

The transmembrane helices assemble into a ligand-binding pocket that represents the structural core of the receptor’s activation mechanism. Upon chemokine engagement, local rearrangements of the 7TM bundle are transmitted inward, especially affecting TM3, TM5, and TM6, which act as “microswitches” that propagate the signal toward intracellular domains [67]. These conformational changes enable the intracellular regions of CCR5 to interact with heterotrimeric G proteins of the Gi/q families, leading to the dissociation of Gαi/q from the Gβγ subunits [68]. This dissociation triggers some signaling events (Figure 1): inhibition of adenylyl cyclase activity (reducing intracellular cAMP levels) [69], activation of phospholipase C-β (PLC-β) with subsequent hydrolysis of PIP2 into IP3 and DAG, mobilization of Ca^2+^ from intracellular stores [70], and activation of protein kinase C (PKC) [71]. In parallel, other signaling routes upregulate the MAPK/ERK cascade, the PI3K/Akt pathway, and members of the Rho family of small GTPases, which collectively orchestrate some cellular processes such as migration, adhesion, survival, and transcriptional regulation [72,73,74].

The intracellular C-terminal tail of CCR5 is enriched in serine and threonine residues that serve as phosphorylation sites for GRKs [75]. Following phosphorylation, these residues recruit β-arrestins, multifunctional adaptor proteins that not only promote receptor internalization through clathrin-coated pits but also with scaffold alternative, G protein-independent signaling pathways [76]. Through this dual role, β-arrestins contribute both to signal desensitization (terminating G protein activation) and to the initiation of noncanonical signaling cascades, including those leading to ERK1/2 activation [77]. CCR5 transport among the plasma membrane, endosomes, and recycling compartments is stringently regulated to balance responsiveness to extracellular chemokines with receptor availability at the cell surface [78].

In terms of evolution and genetics, CCR5 is conserved among vertebrates, underscoring its key role in immune system regulation [79]. The evolutionary conservation of these structural and functional motifs highlights the key role of CCR5 in immune surveillance, leukocyte chemotaxis, and host defense, as variations in these sequences often lead to reduced chemokine binding, disrupted signaling cascades, and increased susceptibility to infectious and inflammatory diseases [80,81]. Nevertheless, genetic variants influence its activity. The most prominent example is the CCR5-Δ32 deletion, a 32-base pair frameshift mutation that results in a truncated, nonfunctional receptor that fails to reach the cell surface [82,83]. Homozygosity for this mutation confers near-complete resistance to HIV-1 infection, as the retrovirus necessitates the CCR5 as a major co-receptor for entry into CD4^+^ T cells and macrophages [84]. Heterozygous individuals exhibit delayed disease progression, reflecting a dose-dependent relationship between CCR5 surface density and viral infectivity [85]. In addition to HIV-1 infection, CCR5 polymorphisms influence susceptibility to other infectious diseases, such as West Nile, SARS-CoV-2, and HBV viruses [86,87,88], as well as in inflammatory and autoimmune disorders characterized by dysregulated chemokine signaling [89,90].

### 2.2. Distribution of the Chemokine CCR5 Receptor

The CCR5 receptor displays a complex and finely tuned distribution pattern across immune and non-immune systems, reflecting its central role in mediating leukocyte trafficking [91], shaping inflammatory microenvironments [92], and influencing host–pathogen interactions [93]. Its expression is neither static nor homogeneous; rather, it is dynamically regulated by cellular activation states, tissue context, cytokine milieu, and pathological conditions [94].

Within the immune system, CCR5 expression is most prominently localized to activated and memory T lymphocytes, with a particularly high prevalence among CD4^+^ T cell subsets exhibiting a Th1-polarized functional phenotype [95,96]. CCR5^+^ CD4^+^ T cells exhibit a significant capacity for directed migration toward sites of antigen presentation and microbial insult, a process orchestrated by chemotactic gradients of the high-affinity ligands (e.g., CCL3, CCL4, and CCL5) [97]. Moreover, several subsets of CD8^+^ T lymphocytes express CCR5, and their receptor-dependent chemotactic responsiveness allows the targeted mobilization of cytotoxic effector functions within virally infected or inflamed tissue microenvironments [98]. This dual presence in both helper and cytotoxic T cells ensures that CCR5 orchestrates coordinated adaptive immune responses, tightly linking antigen recognition with tissue-directed homing [99].

Monocytes and macrophages further exemplify the functional importance of CCR5 distribution. Circulating monocytes, particularly the inflammatory CD14^+^CD16^−^ subset, upregulate CCR5 upon activation and during differentiation into macrophages, enabling their recruitment into tissues where inflammatory mediators are present [100]. Tissue-resident macrophages, such as those in the gut lamina propria [101], the liver [102], and the lung [103], also express CCR5 under pro-inflammatory conditions, enhancing local immune surveillance and contributing to persistent inflammatory signaling pathways. Dendritic cells (DCs), especially immature subsets populating epithelial surfaces, utilize CCR5 not only to migrate into lymphoid structures but also to associate with other immune cells within inflamed tissues [104]. Similarly, NK cells use CCR5 to migrate toward virally infected or neoplastic tissues, thus facilitating the execution of innate immune effector functions [105].

Although historically considered an immune-cell-restricted receptor, CCR5 has been observed in several non-hematopoietic compartments under pathological circumstances. Endothelial cells might express CCR5 during vascular inflammation [106], where receptor activation promotes leukocyte adhesion and transmigration across the endothelium [107]. Similarly, epithelial cells in inflamed mucosal surfaces can transiently upregulate CCR5, possibly serving as amplifiers of immune signaling [108]. In the CNS, CCR5 expression has been distinguished in microglia [109], astrocytes [43], and infiltrating leukocytes during neuroinflammatory events [110], where its presence is linked to glial activation, neuronal damage, and compromise of BBB integrity.

Even within cancer biology, CCR5 has been implicated in a wide spectrum of malignancies, including breast [111], prostate [112], colorectal [113], pancreatic [114], and gastric cancers [115], as well as hematological malignancies such as acute myeloid leukemia [116] and multiple myeloma [117]. Its expression has been linked to tumor-promoting inflammation, metastatic dissemination, and modulation of the tumor microenvironment, suggesting a broad relevance across both solid tumors and hematologic neoplasms [118].

### 2.3. Functional Significance of CCR5 Receptor

#### 2.3.1. Role in Chemotaxis and Immune Cell Trafficking

CCR5-driven chemotaxis plays a key role in shaping adaptive immune responses by directing the migration of CD4^+^ and CD8^+^ T lymphocytes, monocytes, and DCs to sites of inflammation or infection [119]. This redistribution of immune cells ensures that effector populations are concentrated in peripheral tissues where microbial antigens, danger signals, or tissue damage are present [120]. Through directing the activity of Th1 and Th17 subsets, CCR5 signaling promotes cell-mediated immunity, the production of pro-inflammatory cytokines (e.g., IFN-γ, TNF-α, and IL-17), and the clearance of intracellular pathogens [121,122]. In monocytes and DCs, CCR5 activation allows tissue infiltration, where these cells undergo differentiation and antigen presentation, thereby bridging innate and adaptive immune compartments [104,123].

Beyond its role in cell trafficking, CCR5 supports the structural and functional stabilization of immunological synapses between T cells and antigen-presenting cells (APCs) [124]. Mechanistically, CCR5 signaling enhances the affinity of integrins (e.g., integrin α4), thereby strengthening cell–cell adhesion [125]. This supports prolonged T cell-APC interactions, efficient antigen recognition, and sustained TCR signaling, which are crucial for effective clonal expansion and effector differentiation [126]. CCR5-orchestrated cytoskeletal remodeling further reinforces the polarization of signaling complexes and the directional secretion of cytokines and lytic granules toward target cells [127].

In the CNS, CCR5 expression on resident microglia, perivascular macrophages, and infiltrating monocytes regulates leukocyte recruitment during neuroinflammatory conditions [42,43,128]. Under physiological conditions, this contributes to the clearance of pathogens and debris, as well as the resolution of acute insults (e.g., viral encephalitis or traumatic injury) [129]. However, dysregulated CCR5 activation has deleterious effects. Persistent chemokine gradients drive continuous immune cell infiltration, excessive release of ROS, NO, and pro-inflammatory cytokines, ultimately amplifying neuroinflammation and contributing to neuronal dysfunction and loss [130].

#### 2.3.2. CCR5 as a Co-Receptor for HIV-1 Entry

One of the most clinically significant functions of CCR5 lies in its role as a principal co-receptor for HIV-1 [131]. Initially, the HIV-1 envelope glycoprotein gp120 binds to the CD4 receptor on CD4^+^ T cells, macrophages, and DCs. This binding promotes conformational rearrangements in gp120, especially in the V1/V2 region, which exposes the V3 loop. The V3 loop subsequently interacts with the extracellular domains and transmembrane regions of CCR5, mainly the N-terminal sulfated tyrosines and the ECL2 [132,133].

The interaction between gp120 and CCR5 induces further conformational rearrangements that expose the gp41 fusion peptide. Following this, gp41 embeds its fusion domain into the target cell membrane and adopts a six-helix bundle conformation through extensive structural refolding [134]. This conformational change juxtaposes the viral and target cell membranes, driving membrane fusion and the creation of a fusion pore. Through this pore, the viral capsid is delivered into the cytoplasm, thus initiating the HIV-1 replication cycle [135].

#### 2.3.3. CCR5 in Inflammation and Autoimmunity

Beyond its classical role in mediating chemotactic responses, CCR5 is involved in the regulation and fine-tuning of inflammatory processes [39]. Dysregulated CCR5 signaling can precipitate maladaptive immune responses, thereby contributing to the pathogenesis of autoimmune disorders, such as rheumatoid arthritis (RA), multiple sclerosis (MS), and systemic lupus erythematosus (SLE). In these pathologies, CCR5-expressing immune cells perpetuate tissue injury through sustained infiltration, local production of pro-inflammatory cytokines and ROS, collectively amplifying inflammation and promoting chronic tissue damage [136,137,138].

#### 2.3.4. CCR5 in Tissue Repair and Regeneration

Emerging evidence indicates that CCR5 plays a multifaceted role in tissue repair and regeneration, extending beyond its well-characterized functions in immune cell trafficking and inflammatory modulation [106,139]. During the reparative response, CCR5 mediates the directed migration of various reparative cell populations, including macrophages and mesenchymal stem cells (MSCs), to regions of injured tissue. This recruitment is mediated by CCR5 binding to its ligands, which establish chemotactic gradients within the damaged tissue microenvironment [140]. Following recruitment, CCR5-expressing macrophages promote the resolution of inflammation by undergoing polarization from a pro-inflammatory M1 phenotype to an M2 reparative/anti-inflammatory phenotype [141]. M2 macrophages secrete anti-inflammatory cytokines, such as IL-10 and TGF-β, and produce MMPs, thus facilitating remodeling of the ECM [142]. In parallel, CCR5 activation promotes angiogenesis via the production of VEGF, thereby promoting restoration of tissue perfusion and structural integrity [139].

#### 2.3.5. CCR5 in Stem Cell and Hematopoietic Regulation

CCR5 has been increasingly recognized as an important modulator of hematopoietic stem and progenitor cell (HSPC) dynamics [143]. Beyond its well-established role in leukocyte trafficking and immune cell activation, CCR5 contributes to the regulation of migration, homing, and retention of HSPCs within the bone marrow niche [144]. HSPCs reside in specialized microenvironments where interactions with stromal cells, extracellular matrix components, and soluble factors are critical for maintaining stemness, quiescence, and differentiation potential [145]. CCR5 activation shapes these processes by modulating chemotactic signaling and driving cytoskeletal rearrangements that support the directed migration and spatial organization of cells within the niche [146].

Under homeostatic conditions, CCR5 appears to have a relatively modest role compared to other chemokine receptors (e.g., CXCR4). However, its functional importance is markedly amplified under inflammatory conditions [147]. In the context of infection, tissue injury, or subsequent to myeloablative conditioning regimens, CCR5 expression in the HSPC compartment can be upregulated, enhancing their responsiveness to inflammatory chemokines and promoting mobilization from or recruitment back to the bone marrow [148].

Furthermore, CCR5-mediated signaling has been implicated in the crosstalk between HSPCs and the immune system. Pro-inflammatory cytokines can induce CCR5 expression on HSPCs, which in turn allows these cells to sense and migrate toward inflamed tissues [149].

## 3. The Role of CCR5 in the Pathophysiology of Neuropathic Pain

### 3.1. CCR5-Mediated Peripheral Mechanisms in Neuropathic Pain

Peripheral nerve injury constitutes a critical event that initiates a complex cascade of cellular and molecular responses within the PNS, which collectively contribute to the initiation and persistence of neuropathic pain [150]. The initial mechanical or chemical insult results in axonal degeneration, Wallerian degeneration, and demyelination, accompanied by the release of DAMPs, ATP, and pro-inflammatory cytokines from both neuronal and non-neuronal cells [151]. These factors create a highly chemotactic environment that promotes the recruitment and activation of several immune cells, including monocytes, macrophages, neutrophils, and T lymphocytes [152].

Concurrently, CCR5 expression is upregulated on both infiltrating immune cells and resident sensory neurons within the dorsal root ganglia (DRG). The engagement of CCR5 on immune cells not only governs chemotaxis but also triggers intracellular signaling cascades that result in the transcriptional and translational upregulation of numerous inflammatory mediators, such as pro-inflammatory cytokines, prostaglandins, leukotrienes, and ROS [153,154,155]. Collectively, these mediators establish an inflammatory milieu (known as *inflammatory soup*) that potentiates nociceptor sensitization and contributes to peripheral hyperexcitability [156].

On the other hand, CCR5 exerts multifaceted effects on sensory neurons, modulating their immediate excitability and long-term functional plasticity. Although not yet entirely established, CCR5-mediated activation of nociceptors is likely to promote the activation of VGCCs, VGSCs, and TRPs. VGSCs (e.g., Nav1.7, Nav1.8, and Nav1.9) experience transcriptional upregulation and post-translational phosphorylation, strengthening their conductance and lowering the threshold for action potential generation [157]. VGCCs (e.g., Cav2.2 and Cav3.2) are equivalently modulated, increasing intracellular Ca^2+^ influx, which potentiates presynaptic release of neurotransmitters and potentiates abnormal excitability [158]. TRP channels (e.g., TRPV1, TRPA1, and TRPM8) are sensitized via direct phosphorylation by MAPKs or PKA/PKC signaling, enhancing responsiveness to thermal, mechanical, and chemical stimuli [159]. Ultimately, nociceptor activation results in the release of excitatory neurotransmitters onto the spinal cord, driving central sensitization and pain transmission. Primary afferent terminals within the dorsal horn discharge glutamate, substance P, and CGRP, which act on postsynaptic neurons and surrounding glial cells [160]. Moreover, substance P promotes peripheral immune cell recruitment and local inflammation, leading to decreased excitation thresholds in non-nociceptive fibers. This process facilitates aberrant neuronal activation and contributes to peripheral sensitization, thereby amplifying pain perception and driving hyperalgesia and allodynia [161].

Glial cells, including satellite glial cells and Schwann cells in injured nerve fibers, also express CCR5 and are critical mediators of peripheral sensitization following nerve injury [55,162]. Based on investigations conducted with other chemokine receptors (e.g., CCR2 and CX3CR1), CCR5 signaling in these glial cells probably mobilizes intracellular Ca^2+^ and activates PLC-γ and PI3K-Akt pathways, thereby promoting the release of pro-inflammatory cytokines (e.g., TNF-α and IL-1β) and pro-inflammatory mediators (e.g., prostaglandin E2) [163,164]. TNF-α and IL-1β can upregulate the membrane trafficking and surface expression of VGSCs, particularly Nav1.7 and Nav1.8, in DRG neurons [165,166]. Mechanistically, TNF-α binds to TNFR1/2 receptors, leading to activation of downstream signaling cascades such as the p38 MAPK and ERK1/2 pathways [167]. These kinases phosphorylate specific Ser/Thr residues on Nav1.7 and Nav1.8, increasing channel open probability and accelerating recovery from inactivation [168]. IL-1β, through IL-1RI, activates MyD88-dependent signaling, resulting in the activation of IRAK1/4, TRAF6, and NF-κB, thus promoting the transcriptional upregulation of VGSCs [169]. IL-6 and PGE2 modulate several GPCRs expressed in DRG neurons, with IL-6 signaling mediated via its receptor complex (IL-6Rα/gp130) and PGE2 signaling through EP2 and EP4 receptors [170,171]. Activation of these receptors stimulates adenylate cyclase, increasing intracellular cAMP levels and activating PKA. PKA phosphorylates TRP channels, like TRPV1, on cytoplasmic Ser/Thr residues, reducing their activation threshold and promoting channel opening in response to thermal stimuli. Concurrently, Gq/11-coupled EP receptors activate PLC, leading to the generation of DAG and IP_3_, which subsequently activate PKC and mobilize intracellular Ca^2+^ stores. PKC-mediated phosphorylation further sensitizes TRPV1 and other TRP channels, thus enhancing Ca^2+^ influx and contributing to thermal hyperalgesia [172]. NO, produced by inducible nitric oxide synthase (iNOS) in glial cells, together with ROS generated via NADPH oxidase or mitochondrial pathways, modulate the gating properties of multiple ion channels in DRG neurons [173]. NO activates guanylyl cyclase, increasing cGMP levels and stimulating protein kinase G (PKG), which phosphorylates and modifies the activity of VGSCs and K^+^ channels, thus enhancing excitatory currents [174]. Additionally, NO and ROS enhance synaptic glutamate release, further potentiating nociceptive signaling [175,176].

Therefore, CCR5 represents a likely key node in peripheral neuropathic pain mechanisms by integrating chemokine-mediated immune recruitment, glial-neuronal crosstalk, intracellular kinase signaling, ion channel modulation, and transcriptional upregulation of pronociceptive mediators. All of these events establish and maintain a hyperexcitable peripheral nociceptive network that supports the persistence and chronicity of neuropathic pain, making CCR5 a promising target for therapeutic interventions aimed at disrupting maladaptive peripheral sensitization.

### 3.2. CCR5-Mediated Central Mechanisms in Neuropathic Pain

Following peripheral sensitization, nociceptive inputs originating from damaged tissues are transmitted via primary afferent fibers to the dorsal horn of the spinal cord. Upon arrival, these signals initiate various molecular and cellular responses that modulate pain processing [177]. Notably, CCR5 expression is markedly elevated in both dorsal horn neurons and resident microglial populations. This upregulation is temporally correlated with the progression of central sensitization, suggesting a critical modulatory role for CCR5 in the amplification and maintenance of nociceptive signaling [54,178].

**Figure 2:**
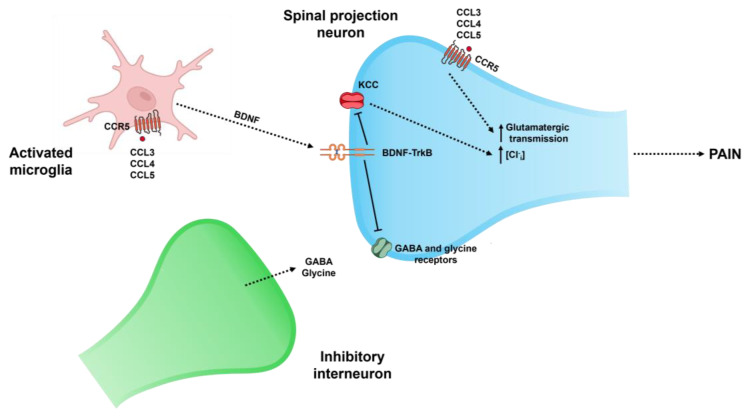
CCR5-mediated microglial modulation of spinal projection neurons in pain signaling. Activation of CCR5 in microglia leads to the release of BDNF. BDNF acts on TrkB receptors in spinal projection neurons, reducing KCC-mediated Cl^−^ extrusion. This results in increased intracellular Cl^−^ and impaired inhibitory neurotransmission through GABA and glycine receptors. Consequently, excitatory glutamatergic transmission is enhanced, promoting pain signaling. Abbreviations: CCR5 (C-C chemokine receptor type 5), CCL3 (chemokine -C-C motif- ligand 3), CCL4 (chemokine -C-C motif- ligand 4), CCL5 (chemokine -C-C motif- ligand 5), BDNF (brain-derived neurotrophic factor), TrkB (tropomyosin receptor kinase B), KCC (K^+^-Cl^−^ cotransporter), [Cl^−^_i_] (intracellular Cl^−^ concentration), and GABA (gamma-aminobutyric acid).

Within the spinal dorsal horn, CCR5 contributes to central sensitization through multiple convergent mechanisms that involve intricate neuroimmune and neuroglial interactions (Figure 2): (1) microglial activation: induction of CCR5 (by CCL3, CCL4, and CCL5) activates intracellular signaling cascades, like the Gi/o protein-dependent inhibition of adenylate cyclase, resulting in decreased cAMP levels, and the downstream modulation of PI3K/Akt and ERK1/2 MAPK pathways [42,179]. All of these processes promote microglial proliferation and phenotypic activation, marked by increased expression of Iba1, CD11b, and MHC-II [180]. Activated microglia release a repertoire of pro-inflammatory mediators that recruit additional immune cells into the spinal cord parenchyma [181]. Importantly, CCR5-driven signaling enhances the release of BDNF, which acts on neuronal TrkB receptors. BDNF-TrkB pathway activation downregulates the neuronal KCC2 cotransporter in dorsal horn projection neurons, resulting in a depolarizing shift in the Cl^−^ reversal potential and thus inhibiting GABAergic and glycinergic transmission [182,183,184]. This dysregulation of the excitatory-inhibitory balance constitutes a key mechanism of central sensitization [185]; (2) synaptic plasticity: CCR5-mediated signaling in dorsal horn neurons regulates activity-dependent synaptic plasticity by modulating the function and trafficking of glutamatergic receptor systems [186]. Upon ligand binding, CCR5 signaling induces pERK, which facilitates NMDA receptor complex activation [187,188]. This fact strengthens NMDA receptor channel conductance and extends channel open time, thus facilitating Ca^2+^ influx [189]. Increased intracellular Ca^2+^ activates signaling molecules like CaMKIV and CREB, which regulate transcription of plasticity-related genes (e.g., c-fos, Arc, and BDNF) [190]. Simultaneously, CCR5 engagement drives the trafficking of GluA1-containing AMPA receptors to the postsynaptic membrane. These molecular modifications consolidate a state of LTP-like plasticity in nociceptive pathways of the dorsal horn [191,192]; (3) neuron-glia feedback loops: neurons within the dorsal horn that express CCR5 respond to chemokine ligands by activating NF-κB and AP-1 transcriptional programs, leading to de novo production of CCL2, CCL5, and other pro-inflammatory mediators [186,193,194]. These chemokines function via autocrine and paracrine mechanisms, thereby further activating CCR5 on adjacent microglia and astrocytes [195]. Moreover, CCR5 signaling modulates presynaptic release machinery through Gβγ subunit interactions with VGCCs, facilitating neurotransmitter release [186]. This fact enhances excitatory drive onto microglia and astrocytes, which respond with further cytokine and chemokine secretion [186,196]. Although the exact relationship remains unclear, astrocytic CCR5 activation likely promotes glutamate release through the system Xc^−^ antiporter and connexin hemichannels, thus intensifying excitotoxic signaling in dorsal horn neurons. This neuroimmune cross-talk establishes a self-reinforcing feedback loop, which is fundamental for the transition from acute nociceptive signaling to chronic neuropathic pain, a state characterized by chronic neuroinflammation and maladaptive synaptic plasticity.

Finally, CCR5 signaling might play a crucial role in supraspinal pain modulation. Current evidence indicates that CCR5 signaling may modulate descending pain control pathways projecting from the periaqueductal gray (PAG), an essential brainstem structure regulating intrinsic pain pathways [197]. Within the PAG, CCR5 expression may influence the balance between excitatory and inhibitory neurotransmission, due to the inhibitory effect exerted on μ-opioid receptors [56].

### 3.3. Interplay Between CCR5 and Other Chemokine Receptors in the Modulation of Neuropathic Pain

CCR5 serves as a central integrator within the chemokine receptor network, orchestrating glial activation, neuronal sensitization, and immune cell trafficking to regulate the onset and development of neuropathic pain [39,42,43,104,106,119,120,121,122,123,124,125,126,127,128,129,130,131,132,133,134,135,136,137,138,139,140,141,142,143,144,145,146,147,148,149]. Its crosstalk with other chemokine receptors establishes an integrated regulatory system that potentiates microglial and astrocytic responses while leukocyte recruitment to sites of neuronal injury [198,199]. This network regulates pro-inflammatory signaling yet may facilitate maladaptive neuroplasticity when disrupted.

CCR2, the primary receptor for CCL2, is integrated with CCR5 signaling, given their co-expression on spinal microglia, astrocytes, DRG neurons, and infiltrating monocytes, where their concomitant activation induces synergistic intracellular pathways [200,201]. CCR5 engagement primes CCR2-dependent pathways such as G-protein-mediated Ca^2+^ influx, MAPK/ERK phosphorylation, and NF-κB nuclear translocation, leading to enhanced production of pro-inflammatory cytokines (such as IL-1β, IL-6, and TNF-α), chemokines (e.g., CCL3 and CCL5), while CCR2 activation reciprocally regulates CCR5 surface expression and ligand sensitivity, orchestrating a feed-forward loop that sustains microglial reactivity and central sensitization [202]. Indeed, some studies support the crosstalk between CCR2 and CCR5. In CCR5-lacking mice, CCR5 deficiency promotes LPS-induced astrogliosis, Aβ plaque deposition, and memory impairment due to increased CCR2 expression in the CNS [203]. Moreover, the involvement of CCR2 and CCR5 has been shown in preclinical models of obesity-induced hypersensitivity and diabetic neuropathy, where administration of dual CCR2/CCR5 antagonists led to pain reduction [204].

Beyond CCR2, CCR5 also shows functional crosstalk with CXCR3, a receptor for the IFN-inducible chemokines CXCL9, CXCL10, and CXCL11, which mediates T-cell recruitment to the spinal cord and DRG [205]. CCR5 influences CXCR3 signaling indirectly by modulating the local chemokine milieu, upregulating CXCR3 ligand expression in resident glia and infiltrating lymphocytes, and potentially co-localizing with CXCR3 on microglia, thereby coordinating downstream signaling several cascades including PI3K/Akt, ERK1/2, and JAK/STAT pathways that enhance glial activation, sustain pro-inflammatory cytokine release, and facilitate the persistence of neuropathic hyperalgesia [206].

On the other hand, CCR5 displays functional redundancy with CCR1 and CCR3, supporting overlapping ligand binding, mainly with CCL5 and CCL3, which can compensate for CCR5 blockade and maintain chemokine-mediated microglial activation and leukocyte recruitment [186]. This redundancy underscores the chemokine signaling network and accounts for the partial analgesic effects seen with CCR5 antagonists.

CCR5 interacts with other chemokine receptors to regulate inflammatory signaling, but dysregulation of this network can induce maladaptive neuroplasticity when it becomes dysregulated, driving chronic neuropathic pain marked by allodynia, hyperalgesia, and spontaneous pain [207]. Multi-target strategies concurrently inhibiting CCR5 and CCR2, CXCR3, or CX3CR1 suppress compensatory signaling, attenuate glial activation, and control maladaptive neuroinflammation without inducing immune suppression.

## 4. CCR5 Inhibition: Emerging Approaches for Analgesia

### 4.1. Evidence from Animal Models of Neuropathic Pain

Emerging evidence increasingly implicates CCR5 as a critical regulator of neuroinflammatory pathways that contribute to the initiation and maintenance of neuropathic pain. Preclinical studies using diverse rodent models of neuropathic pain, like CCI, PSNL, CIPN, and DPN, have shown that modulation of CCR5 attenuates pathological pain behaviors. Genetic deletion of CCR5, pharmacological antagonism with small molecules or monoclonal antibodies, and targeted knockdown via RNA interference all result in significant reductions in mechanical allodynia, thermal hyperalgesia, and spontaneous pain behaviors. The following table provides a comprehensive overview of recent preclinical investigations evaluating CCR5-targeted interventions across diverse neuropathic pain models.

Table 1 shows an overview of the representative CCR2/CCR5-targeting compounds, while Table 2 summarizes their pharmacological characteristics, clinical indications, mechanisms of action, pharmacokinetic profiles, and clinical development status, and Table 3 presents the adverse effects reported following their administration. 

### 4.2. Translational Potential and Early Clinical Findings

Clinical evidence for CCR5 blockade in neuropathic pain remains at an early stage. Maraviroc has received FDA approval for long-term use in HIV-1 infection [215] (Table 2), with a generally acceptable safety profile that includes routine hepatotoxicity monitoring and careful consideration of concomitant medications due to CYP3A4-mediated drug–drug interactions [216]. However, several pharmacological and clinical limitations restrict its suitability for chronic pain indications. Idiosyncratic hepatotoxicity, although uncommon, has been documented and necessitates ongoing hepatic function surveillance during long-term treatment (Table 3) [226]. Moreover, maraviroc displays relatively low oral bioavailability, largely attributable to extensive first-pass hepatic metabolism, which complicates dose optimization and contributes to interindividual variability in systemic exposure [229]. An additional limitation is its reduced permeability across the BBB. Although maraviroc exerts immunomodulatory effects in peripheral tissues, its constrained CNS penetration raises concerns regarding adequate engagement of CCR5 within spinal and supraspinal regions that mediate pain processing, where CCR5-driven neuroinflammatory cascades are central to the onset and development of neuropathic pain [230]. Consequently, attaining therapeutically meaningful CCR5 inhibition in the CNS with maraviroc might require elevated systemic exposure, thus increasing the risk of hepatotoxicity and unfavorable pharmacokinetic interactions.

These limitations have driven intensified investigation into alternative CCR5 blockade strategies. Monoclonal antibodies targeting CCR5 have emerged as promising candidates due to high target specificity, reduced hepatic metabolism, and extended systemic half-life, potentially enabling more consistent receptor occupancy. Unlike small-molecule antagonists, specific monoclonal antibodies exhibit increased pharmacokinetic properties and, when optimally engineered, may enable more efficient modulation of neuroimmune signaling [231]. While randomized controlled trials of maraviroc in neuropathic pain have yet to be conducted, its thoroughly defined clinical pharmacology profile supports assessment in proof-of-concept studies and reinforces the potential of next-generation CCR5 inhibitors, like monoclonal antibodies, as promising therapeutic candidates for chronic pain.

Monoclonal antibodies directed against CCR5, including leronlimab (PRO-140), have been tested in clinical populations like HIV-1, oncology, and COVID-19 [232,233,234]. These studies confirm sustained receptor occupancy, predictable pharmacokinetics, and a favorable safety profile. Although no neuropathic pain-specific clinical data are currently available, the immunomodulatory actions of leronlimab align well with mechanistic pathways implicated in neuroinflammatory pain. The long-lasting therapeutic effect and receptor interaction of monoclonal antibodies could be helpful in chronic pain diseases requiring suppression of immune-neuronal signaling [235].

## 5. Future Perspectives

The evolving understanding of CCR5’s role in neuropathic pain opens multiple avenues for future research and therapeutic development. A key focus is the identification of predictive biomarkers to enable precise patient stratification [236]. By elucidating molecular signatures, chemokine profiles, or genetic polymorphisms associated with enhanced CCR5 signaling, researchers may be able to determine which patient populations are most likely to benefit from CCR5-targeted interventions. This precision medicine approach has the potential to optimize clinical trial design and improve therapeutic outcomes [237].

Another promising avenue is the investigation of combinatorial strategies with existing analgesics. Integrating CCR5 antagonists with conventional pharmacotherapies (such as opioids) might produce synergistic effects and enhance analgesic efficacy while reducing doses and minimizing adverse events [238]. Systematic preclinical and translational studies are required to identify optimal combinations, dosing regimens, and timing of administration to maximize therapeutic benefit and minimize toxicity.

The development of next-generation CCR5 modulators is equally important. Current pharmacological agents, while effective, may have limitations in selectivity, pharmacokinetics, or tissue penetration [239]. Rational drug design approaches, including structure-based improvement [240], allosteric modulators [241], and monoclonal antibodies [242], could yield compounds with enhanced receptor specificity, improved CNS penetration, and prolonged half-life, thereby improving clinical efficacy and safety profiles.

Moreover, advanced translational models should be employed to bridge the gap between preclinical findings and clinical application. Humanized mouse models [243], patient-derived organoids [244], and high-throughput screening platforms [245] could provide insights into CCR5 biology in human tissues, helping to refine therapeutic strategies. Additionally, longitudinal clinical studies will be crucial to evaluate long-term safety, efficacy, and potential off-target effects of CCR5-targeted therapies [208].

Finally, a multidisciplinary approach integrating immunology, neuroscience, pharmacology, and clinical pain research will be fundamental for accelerating the translation of CCR5 antagonists from bench to bedside [246]. Through these concerted efforts, CCR5-targeted therapies hold the potential not only to ameliorate neuropathic pain but also to redefine mechanistically informed treatment paradigms for patient’s refractory to conventional analgesics.

## 6. Conclusions

CCR5 has emerged as a critical mediator of neuroinflammatory processes underlying the development and maintenance of neuropathic pain. Preclinical evidence from diverse rodent models demonstrates that CCR5 contributes to central sensitization through activation of glial cells and several neuronal populations, as well as peripheral sensitization through recruitment of immune cells to injured nerves and modulation of DRG neurons. Pharmacological antagonism, genetic deletion, or targeted knockdown of CCR5 alleviates mechanical allodynia, thermal hyperalgesia, and spontaneous pain behaviors, highlighting its pivotal role in pain pathophysiology.

These findings are encouraging and justify continued investigation of CCR5 signaling in nociceptive modulation; however, major gaps in mechanistic insight and translational feasibility persist. Some referenced preclinical studies present methodological weaknesses that limit interpretability and external validity. Common limitations include small sample sizes, huge reliance on rodent models without validation in higher-order species, and inadequate behavioral controls or standardized pain assessment protocols. As a result, reproducibility and internal validity are uncertain, and extrapolation to human pain physiology remains limited.

To address these issues, future research must implement more rigorous experimental designs, including appropriate statistical power, randomization, blinding, and validated behavioral assays. Translational studies are needed to define dosing strategies, pharmacokinetic and pharmacodynamic profiles, and long-term safety, particularly regarding the immunomodulatory risks of chronic CCR5 inhibition. Ultimately, randomized controlled trials in humans will be essential to establish therapeutic efficacy across diverse pain conditions, including neuropathic, inflammatory, and cancer-related pain.

CCR5 antagonists hold therapeutic value by targeting neuroimmune pathways and introducing a novel intervention strategy for neuropathic pain syndromes that do not respond to conventional analgesic therapies. Compounds including maraviroc and experimental monoclonal antibodies have demonstrated efficacy in preclinical models, providing a strong rationale for clinical translation. Given the multifactorial nature of chronic pain, future investigations should also evaluate combinatorial or synergistic approaches that integrate CCR5-targeted therapies with standard analgesics (e.g., opioids, gabapentinoids, or anti-inflammatory agents) to enhance efficacy while minimizing adverse effects and tolerance.

In summary, CCR5 represents a promising molecular target for next-generation analgesics; however, clinical implementation will require methodologically rigorous, interdisciplinary research that bridges preclinical findings with patient-centered outcomes.

## Figures and Tables

**Table 1:** CCR5 antagonists in preclinical neuropathic pain models. Abbreviations: CCI (chronic constriction injury), CCR5 (C-C chemokine receptor type 5), i.t. (intrathecal injection), p38 MAPK (p38 mitogen-activated protein kinase), ERK1/2 (extracellular signal-regulated kinases 1 and 2), NF-κB (nuclear factor kappa-light-chain-enhancer of activated B cells), STAT3 (signal transducer and activator of transcription 3), DRG (dorsal root ganglion), IL-1β (interleukin 1 beta), IL-18 (interleukin 18), IL-6 (interleukin 6), NOS2 (nitric oxide synthase 2), IL-1RA (Interleukin 1 receptor antagonist), IL-18BP (Interleukin 18 binding protein), IL-10 (Interleukin 10), Iba-1 (ionized calcium-binding adapter molecule 1), GFAP (glial fibrillary acidic protein), qRT-PCR (quantitative real-time polymerase chain reaction), CCL3 (chemokine (C-C motif) ligand 3), CCL4 (chemokine (C-C motif) ligand 4), CCL5 (chemokine (C-C motif) ligand 5), CCL7 (chemokine (C-C motif) ligand 7), DPN (diabetic peripheral neuropathy), mRNA (messenger RNA), STZ (streptozocin), CCL8 (chemokine (C-C motif) ligand 8), CCL12 (chemokine (C-C motif) ligand 12), PSNL (partial sciatic nerve ligation), DAPTA (D-Ala1-peptide T-amide), and PIPN (paclitaxel-induced peripheral neuropathy).

Neuropathic Pain Model	Compound	Observed Outcomes	Proposed Mechanisms	References
CCI	Maraviroc	Chronic i.t. administration of maraviroc attenuated neuropathic pain symptoms and elevated the nociceptive threshold approximately 60 min post-administration on days 3 and 7 following CCI	Maraviroc suppressed phosphorylated p38 MAPK, ERK1/2, and NF-κB expression while enhancing STAT3 in the spinal cord and DRG Maraviroc reduced classical pro-nociceptive markers (IL-1β, IL-18, IL-6, and NOS2) and upregulated anti-nociceptive markers (IL-1RA, IL-18BP, and IL-10) in the spinal cord	[54]
CCI	Maraviroc	Chronic i.t. administration of maraviroc alleviated neuropathic pain symptoms on day 7 post-CCI	Maraviroc decreased Iba-1 and GFAP protein levels and restored CCR5 expression altered by CCI in the spinal cord and DRG qRT-PCR showed that CCR5 and its pro-nociceptive ligands (CCL3, CCL4, and CCL5) were upregulated after nerve injury, while maraviroc attenuated these increases	[208]
CCI	Maraviroc Cenicriviroc	Reduction in neuropathic pain outcomes	Maraviroc reduced CCI-induced increases in CCL4 within the spinal cord and selectively decreased CCL5 expression in the DRG In contrast, cenicriviroc exerted a broader effect, reducing CCL2, CCL3, CCL4, and CCL7 in the spinal cord, and CCL2, CCL3, CCL4, CCL5, and CCL7 in the DRG	[209]
DPN	Cenicriviroc	A single dose of cenicriviroc produced comparable analgesia in male and female mice Repeated cenicriviroc elicited the most robust and sustained antinociceptive effect, reducing STZ-induced hypersensitivity without tolerance	In male mice, mRNA levels of CCL2, CCL5, and CCL7 were upregulated, whereas female mice exhibited additional upregulation of CCL8 and CCL12	[55]
PSNL	Maraviroc	i.t. administration of maraviroc following PSL significantly attenuated mechanical allodynia exclusively in male mice	Not defined	[47]
CCI	TAK-220 AZD-5672	Single intrathecal administration of TAK-220 and AZD-5672 dose-dependently reduced pain-related behaviors following CCI	Not defined	[210]
CCI	RAP-103	Oral administration of RAP-103 robustly attenuated nerve injury-induced mechanical and thermal hypersensitivity	RAP-103 treatment slightly reduced GFAP expression, while Iba-1-positive cells in the spinal cord exhibited smaller cell bodies, elongated fine processes, and markedly reduced Iba-1 staining	[211]
PSNL	DAPTA (Adaptavir)	DAPTA administration prevented the development of tactile allodynia and thermal hyperalgesia	Not defined	[212]
PIPN	Maraviroc	Administration of the CCR5 antagonist maraviroc attenuates the development of neuropathic pain-related nociceptive behaviors	Not defined	[213]

**Table 2:** Comparative pharmacological characterization of CCR5 and dual CCR2/CCR5 antagonists discussed above. Abbreviations: CCR5 (C-C chemokine receptor type 5), HIV-1 (human immunodeficiency virus type 1), FDA (Food and Drug Administration), CCR2 (C-C chemokine receptor type 2), MASH (metabolic dysfunction-associated steatohepatitis), and PK (pharmacokinetics).

Compound	Target	Indications	Mechanisms of Action	Pharmacokinetics	Clinical Status	References
Maraviroc	CCR5 antagonist	HIV-1	Blocks CCR5 to inhibit HIV-1 entry and immune cell trafficking	Oral administration t½ 14–18 h Activation of CYP3A4	FDA- approved	[214,215,216]
Cenicriviroc	CCR2/CCR5 antagonist	MASH Fibrosis HIV-1	Blocks CCR2 and CCR5- associated inflammatory pathways	Oral administration t½ 22–42 h Poor CYP activation	Phase 3 discontinued	[217,218,219]
TAK-220	CCR5 antagonist	HIV-1	Competitive CCR5 inhibitor	Good oral bioavailability	Not tested	[220]
AZD-5672	CCR5 antagonist	Autoimmune diseases	Blocks CCR5-mediated T-cell trafficking	Oral administration Limited PK data	Phase 2	[221,222]
RAP-103	CCR2/CCR5 antagonist	Severe psoriasis	Reduces macrophage activation	Oral administration Limited PK data	Phase 2	[223] [NCT07204639]
DAPTA (Adaptavir)	CCR5 antagonist	Neuroinflammation HIV-1	Blocks CCR5 and reduces microglial activation	Intranasal or Injectable drug delivery	Not tested	[224,225]

**Table 3:** Adverse effect profile of CCR5 antagonists. Abbreviations: CCR5 (C-C chemokine receptor type 5), CCR2 (C-C chemokine receptor type 2), ALT (alanine aminotransferase), and AST (aspartate aminotransferase).

Compound	Target	Side Effects	References
Maraviroc	CCR5 antagonist	Gastrointestinal symptoms: diarrhea, nausea, and hepatotoxicity Neurological symptoms: headache, dizziness and insomnia Respiratory symptoms: cough Musculoskeletal issues: muscle spasms and back pain Dermatological problems: rash General symptoms: weakness	[226,227]
Cenicriviroc	CCR2/CCR5 antagonist	Gastrointestinal symptoms: diarrhea, nausea, constipation, and abdominal pain Respiratory symptoms: nasopharyngitis and influenza Dermatological problems: rash General symptoms: fatigue, arthralgia, and headache	[228]
AZD-5672	CCR5 antagonist	Gastrointestinal symptoms: nausea, diarrhea, abdominal pain, and dyspepsia Respiratory issues: nasopharyngitis, sinusitis, and bronchitis Dermatological problems: rash and pruritus General symptoms: headache, fatigue, and dizziness Laboratory changes: transient neutropenia and increases in liver enzymes (ALT/AST)	[221]
RAP-103	CCR2/CCR5 antagonist	Not published	[NCT07204639]

## Data Availability

No new data were created or analyzed in this study. Data sharing is not applicable to this article.

## Abbreviations

The following abbreviations are used in this manuscript:

[Cl^−^_i_]	Intracellular Cl^−^ concentration
7TM	Seven transmembrane
Akt	Protein kinase B
ALT	Alanine aminotransferase
AMPA	α-amino-3-hydroxy-5-methyl-4-isoxazolepropionic acid
AP-1	Activator protein 1
APC	Antigen-presenting cell
Arc	Activity-regulated cytoskeleton-associated protein
AST	Aspartate aminotransferase
ATP	Adenosine triphosphate
BBB	Blood–brain barrier
BDNF	Brain-derived neurotrophic factor
c-fos	Cellular oncogene fos
Ca^2+^	Calcium ion
CaMK	Calcium/calmodulin-dependent protein
CaMKIV	Calcium/calmodulin-dependent protein kinase IV
cAMP	Cyclic adenosine monophosphate
cGMP	Cyclic guanosine monophosphate
Cav2.2	Voltage-gated calcium channel subtype 2.2
CCI	Chronic constriction injury
CCL12	Chemokine (C-C motif) ligand 12
CCL2	Chemokine (C-C motif) ligand 2
CCL3	Chemokine (C-C motif) ligand 3
CCL4	Chemokine (C-C motif) ligand 4
CCL5	Chemokine (C-C motif) ligand 5
CCL7	Chemokine (C-C motif) ligand 7
CCL8	Chemokine (C-C motif) ligand 8
CCR1	C-C chemokine receptor type 1
CCR2	C-C chemokine receptor type 2
CCR3	C-C chemokine receptor type 3
CCR5	C-C chemokine receptor type 5
CD11b	Cluster of differentiation 11b
CD14	Cluster of differentiation 14
CD16	Cluster of differentiation 16
CD4	Cluster of differentiation 4
CD8	Cluster of differentiation 8
CGRP	Calcitonin gene-related peptide
Cl^−^	Chloride ion
CNS	Central nervous system
CREB	cAMP response element-binding protein
CXCL9	C-X-C motif chemokine ligand 9
CXCL10	C-X-C motif chemokine ligand 10
CXCL11	C-X-C motif chemokine ligand 11
CXCR3	C-X-C chemokine receptor type 3
CX3CR1	C-X3-C motif chemokine receptor 1
DAG	Diacylglycerol
DAMP	Damage-associated molecular pattern
DAPTA	D-Ala1-peptide T-amide
DC	Dendritic cell
DPN	Diabetic peripheral neuropathy
DRG	Dorsal root ganglia
ECL1	Extracellular loop 1
ECL2	Extracellular loop 2
ECL3	Extracellular loop 3
ECM	Extracellular matrix
EP2	Prostaglandin E2 receptor subtype 2
EP4	Prostaglandin E2 receptor subtype 4
ERK	Extracellular signal-regulated kinase
ERK1/2	Extracellular signal-regulated kinases 1 and 2
FDA	Food and Drug Administration
GABA	Gamma-aminobutyric acid
GFAP	Glial fibrillary acidic protein
GluA1	Glutamate receptor subunit A1 (AMPA receptor subunit)
gp120	Glycoprotein 120
G0p130	Glycoprotein 130
gp41	Glycoprotein 41
GPCR	G protein-coupled receptor
GRK	G protein-coupled receptor kinase
Gαi	G protein alpha i subunit
Gαq	G protein alpha q subunit
Gβγ	G protein beta-gamma subunits
Gq/11	G protein subtypes q and 11
HBV	Hepatitis B virus
HIV-1	Human immunodeficiency virus type 1
i.t.	Intrathecal injection
IASP	International Association for the Study of Pain
Iba1	Ionized calcium-binding adapter molecule 1
ICAM-1	Intercellular adhesion molecule 1
ICL1	Intracellular loop 1
ICL2	Intracellular loop 2
ICL3	Intracellular loop 3
IFN-γ	Interferon gamma
IL-1RI	Interleukin-1 receptor type I
IL-10	Interleukin 10
IL-17	Interleukin 17
IL-18	Interleukin 18
IL-18BP	Interleukin 18 binding protein
IL-1β	Interleukin 1 beta
IL-1RA	Interleukin 1 receptor antagonist
IL-6	Interleukin 6
IL-6Rα	Interleukin-6 receptor alpha
iNOS	Inducible nitric oxide synthase
IP3	Inositol 1,4,5-trisphosphate
IRAK1/4	Interleukin-1 receptor-associated kinases 1 and 4
JAK	Janus kinase
K^+^	Potassium ion
KCC	Potassium chloride cotransporter
KCC2	Potassium chloride cotransporter 2
LTP	Long-term potentiation
M1	Pro-inflammatory microglial phenotype
M2	Anti-inflammatory microglial phenotype
MAPK	Mitogen-activated protein kinase
MASH	Metabolic dysfunction-associated steatohepatitis
MHC-II	Major histocompatibility complex class II
MIP-1α	Macrophage inflammatory protein 1 alpha
MIP-1β	Macrophage inflammatory protein 1 beta
MMP	Matrix metalloproteinase
MS	Multiple sclerosis
MSC	Mesenchymal stem cell
MyD88	Myeloid differentiation primary response 88
mTOR	Mammalian target of rapamycin
Nav1.3	Voltage-gated sodium channel subtype 1.3
Nav1.7	Voltage-gated sodium channel subtype 1.7
Nav1.8	Voltage-gated sodium channel subtype 1.8
NADPH	Nicotinamide adenine dinucleotide phosphate
NF-κB	Nuclear factor kappa-light-chain-enhancer of activated B cells
NGF	Nerve growth factor
NK	Natural killer
NMDA	N-methyl-D-aspartate
NO	Nitric oxide
NOS2	Nitric oxide synthase 2
p38 MAPK	p38 mitogen-activated protein kinase
pERK	Phosphorylated extracellular signal-regulated kinase
PGE2	Prostaglandin E2
PI3K	Phosphoinositide 3-kinase
PI3K-β	Phosphatidylinositol 3-kinase beta
PI3K-γ	Phosphatidylinositol 3-kinase gamma
PIP2	Phosphatidylinositol 4,5-bisphosphate
PIPN	Paclitaxel-induced peripheral neuropathy
PK	Pharmacokinetics
PKA	Protein kinase A
PKB	Protein kinase B
PKC	Protein kinase C
PKG	Protein kinase G
PLC-β	Phospholipase C beta
PLC-γ	Phospholipase C gamma
PNS	Peripheral nervous system
PRR	Pattern recognition receptor
PSNL	Partial sciatic nerve ligation
qRT-PCR	Quantitative real-time Polymerase Chain Reaction
RA	Rheumatoid arthritis
RANTES	Regulated upon activation, normal T cell expressed and secreted
ROS	Reactive oxygen species
SARS-CoV-2	Severe acute respiratory syndrome coronavirus 2
Ser	Serine
SLE	Systemic lupus erythematosus
STAT	Signal transducer and activator of transcription
STAT3	Signal transducer and activator of transcription 3
STZ	Streptozotocin
TCR	T cell receptor
TGF-β	Transforming growth factor beta
Th1	T helper 1 cell
Th17	T helper 17 cell
Thr	Threonine
TM3	Transmembrane helix 3
TM5	Transmembrane helix 5
TM6	Transmembrane helix 6
TNF-α	Tumor necrosis factor alpha
TNFR1	Tumor necrosis factor receptor 1
TNFR2	Tumor necrosis factor receptor 2
TRAF6	TNF receptor-associated factor 6
TrkB	Tropomyosin receptor kinase B
TRP	Transient receptor potential channel
TRPA1	Transient receptor potential ankyrin 1
TRPM8	Transient receptor potential melastatin 8
TRPV1	Transient receptor potential vanilloid 1
V1	Variable region 1 of gp120
V2	Variable region 2 of gp120
V3	Variable region 3 of gp120
VEGF	Vascular endothelial growth factor
VGCC	Voltage-gated calcium channel
VGSC	Voltage-gated sodium channel
Xc^−^	Cystine/glutamate antiporter
